# Quasi-two-dimensional perovskite light emitting diodes for bright future

**DOI:** 10.1038/s41377-021-00528-3

**Published:** 2021-04-19

**Authors:** Jin-Wook Lee, Nam-Gyu Park

**Affiliations:** 1 SKKU Advanced Institute of Nanotechnology (SAINT) and Department of Nanoengineering, Sungkyunkwan University, Suwon, 16419 Republic of Korea; 2 School of Chemical Engineering, Sungkyunkwan University, Suwon, 16419 Republic of Korea

**Keywords:** Inorganic LEDs, Quantum dots

## Abstract

The fundamentals, promise and challenges of metal halide quasi-two-dimensional (quasi-2D) perovskites for a next generation emitter in light emitting diode devices are systematically reviewed.

Display technologies have served as an effective medium to deliver visual information to people in the Information Age. Since the development of cathode ray tube (CRT) in 1897, the display technology has continuously evolved in seeking better resolution, brightness, and color reproducibility with the longer lifetime and lower production cost, resulting in the development of light emitting diodes (LEDs) and liquid crystal displays (LCDs). With upcoming 4^th^ industrial revolution, displays are becoming ubiquitous across a variety of electronic and functional devices with unconventional form factors.

Among various display technologies, LED technologies have continued to improve, and is recently undertaking the market share that was previously dominated by the LCDs. In particular, the performance and lifetime of organic light emitting diodes (OLEDs) developed in 1987 has been greatly improved with additional advantageous features^1^; thin and flexible devices with a high resolution, leading to great success in commercialization. Nevertheless, the OLEDs still face several challenges such as a broad emission bandwidth hindering color purity, limited brightness due to low carrier transport capability and exciton recombination, and short operational lifetimes of the device. As a result, consistent efforts have been devoted for development of alternative emitter materials for the next-generation display technology. Metal halide perovskites (MHPs) have been under spotlight in the field of optoelectronic devices since the first development of solid-state MHP solar cells in 2012^2^. The MHPs feature superior optoelectronic properties beneficial for application in LED devices, such as high photoluminescence quantum yields (PLQYs), a narrow emission bandwidth, and tunable bandgap. Since the first demonstration of MHP LEDs (PeLEDs) by Tan et al. in 2014^3^, the PeLEDs have attracted significant research interests leading to rapid enhancement in its performance to reach a record external quantum efficiency (EQE) above 20%^4,5^.

The prototypical three-dimensional (3D) ABX_3_ MHPs used for a photovoltaic application have high dielectric constants (>10) and low exciton binding energies in the order of few tenths of milli electron volts^6,7^. Consequently, coulombic interaction between electrons and holes are effectively screened, resulting in long diffusion lengths and lifetimes of free carriers^8,9^. While these properties are beneficial for facilitating charge collection in solar cells, it hinders effective radiative recombination of the injected charge carriers in the PeLEDs. The diffusive charge carriers in polycrystalline MHP thin films can be trapped at prevailing defect states to subsequently undergo non-radiative recombination to reduce radiative efficiency of the films and devices. Therefore, appropriate management of carrier recombination dynamics was found to be essential to achieve high-performance PeLEDs.

In attempt to promote radiative recombination, nanocrystalline films of nano grains or crystals are fabricated^10,11^. Confinement of charge carriers in the nano-sized crystals accelerates the radiative recombination of charge carriers resulting in distinct enhancement in the EQE of the PeLEDs. However, reducing the size of crystals inevitably creates abundant surface states which limits performance and stability of the PeLED^12^. Another promising approach is to utilize quasi-two-dimensional (quasi-2D) MHPs^13^. The quasi-2D MHPs consist of alternating planes of corner sharing octahedra (3D MHPs) and bulky organic cations as shown in Fig. 1. Such a structural feature enables formation of the inherent quantum well structure with a large exciton binding energy where the width of the quantum well can be tuned by varying the number of 3D MHPs layers^14^. A publication by Zhang et al. now presents a comprehensive review on inherent properties of quasi-2D MHPs materials as well as methodologies for synthesizing high-quality quasi-2D MHPs with targeted properties for realizing high performance LEDs. Various approaches to achieve color-pure emission of quasi-2D MHPs thin films are presented, which is followed by introduction of strategies for optimization of the LED devices based on the films. Performance metrics of the reported PeLEDs based on the quasi-2D MHPs are summarized where the record EQE has exceeded 20%^15^.

**Fig. 1:**
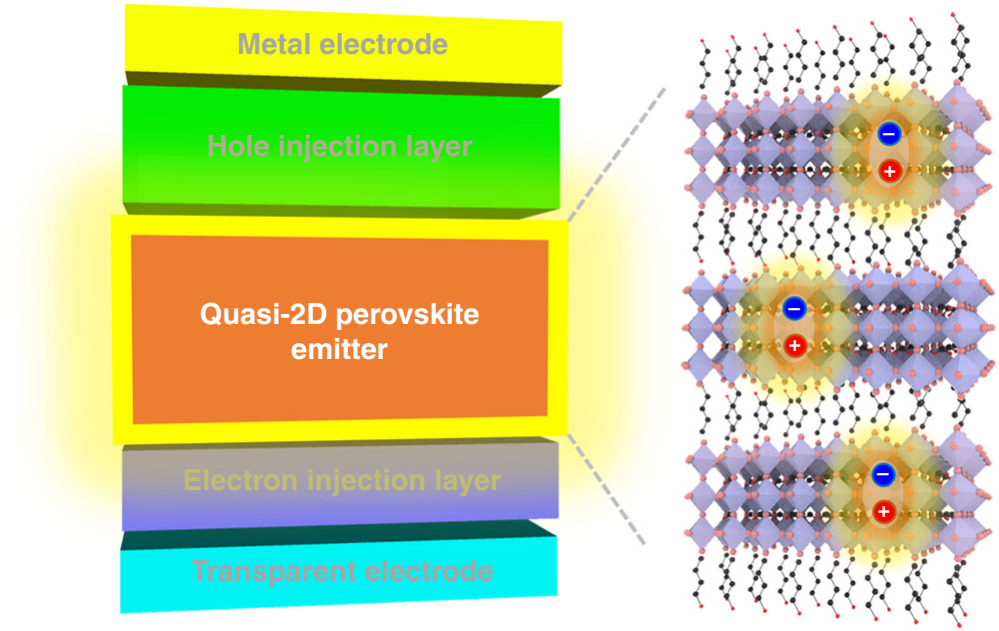
Perovskite light emitting diodes (LEDs) based on quasi-two-dimensional (2D) perovskites. A schematic device architecture of the LEDs (left) and crystal structure of the quasi-2D perovskites with the injected charges confined within three-dimensional perovskite slabs (right)

Despite the rapid improvement in device performance, however, there are still many challenges toward a practical use of the PeLEDs based on quasi-2D MHPs such as environmental toxicity of Pb-based MHPs, limitation in large-scale fabrication, relatively inferior performance of blue and red devices, and poor operational stability of the devices. As suggested by Zhang et al. extensive exploration of novel quasi-2D MHPs as well as a mechanistic understanding of crystallization kinetics will be required to resolve these issues. This seems to be a forthcoming research direction in the field. On the other hand, there are opportunities for broader applications of the quasi-2D MHPs beyond conventional LEDs such as wearable electronics and foldable displays, and lasing devices.
